# Indoor Navigation Systems for Visually Impaired Persons: Mapping the Features of Existing Technologies to User Needs

**DOI:** 10.3390/s20030636

**Published:** 2020-01-23

**Authors:** Darius Plikynas, Arūnas Žvironas, Andrius Budrionis, Marius Gudauskis

**Affiliations:** 1 Department of Business Technologies and Entrepreneurship, Vilnius Gediminas Technical University, 10223 Vilnius, Lithuaniaandrius.budrionis@gmail.com (A.B.); marius.gudauskis@vgtu.lt (M.G.); 2 Norwegian Centre for E-health Research, University Hospital of North Norway, 9019 Tromsø, Norway

**Keywords:** visually impaired, blind, indoor navigation, object detection and recognition, electronic travelling aid, assistive device

## Abstract

Currently, several outdoor navigation and orientation electronic traveling aid (ETA) solutions for visually impaired (VI) people are commercially available or in active development. This paper’s survey of blind experts has shown that after outdoor navigation, the second most important ETA feature for VI persons is indoor navigation and orientation (in public institutions, supermarkets, office buildings, homes, etc.). VI persons need ETA for orientation and navigation in unfamiliar indoor environments with embedded features for the detection and recognition of obstacles (not only on the ground but also at head level) and desired destinations such as rooms, staircases, and elevators. The development of such indoor navigation systems, which do not have Global Positioning System (GPS) locational references, is challenging and requires an overview and evaluation of existing systems with different navigation technologies. This paper presents an evaluation and comparison of state-of-the-art indoor navigation solutions, and the research implications provide a summary of the critical observations, some insights, and directions for further developments. The paper maps VI needs in relation to research and development (R&D) trends using the evaluation criteria deemed most important by blind experts.

## 1. Introduction

According to October 2018 figures from the World Health Organization (WHO) [1], there are approximately 1.3 billion people with some form of vision impairment globally, 36 million of which are blind. The authors [2] forecast that there will be 38.5 million blind people by 2020, and 115 million blind people by 2050. Naturally, an increasing number of people will need navigation assistance in the future.

Vision is considered to be one of the most important human senses. It plays an essential role in allowing people to understand the environment, find a desired goal, and determine the correct path in unknown environments. A lack of vision affects personal and professional relationships. It makes a significant difference in the performance of daily life routines. Globally, there are a number of researchers and high-level engineers who are developing electronic traveling aid (ETA) solutions to help the blind and visually impaired (VI) to better orientate and navigate [3]. However, indoor navigation in unknown environments is still the most important task for VI persons, because due to a weak Global Positioning System (GPS) signal [4], some other special techniques or technologies are needed [5].

Admittedly, at the early stages of the development of a new ETA system, it is crucial to review, compare, and evaluate existing technological solutions. The review of publications from the last five years revealed just a few surveys of indoor positioning and navigation studies. The authors review indoor navigation systems with a focus on aspects of indoor positioning techniques [6] and indoor positioning technologies [7]. Here, positioning techniques applied in indoor positioning systems include signal properties and positioning algorithms. Tapu et al. [3] present a survey of indoor/outdoor wearable/assistive devices and provide a critical presentation of each system while emphasizing their strengths and limitations. The authors also present a classification of wearable/assistive devices using both qualitative and quantitative measures of evaluation. Localization metrics and algorithms such as distance and phase estimation are provided in Mahida et al. [8].

Generalized system architectures for sensor-, computer vision-, and smartphone-based ETAs were presented in a literature review by Islam et al., providing some details on how the components of these system work together [9]. Chronological development in the field was also recently reviewed, showing how ETAs progressed from the early 1960s to the current days [10]. While some authors choose to set their main focus on reviewing various solutions addressing the components of navigation process (for instance, obstacle detection and avoidance [11,12]), the others take a more holistic approach and assess the pros and cons of assistive technology for navigating in unknown surroundings [13,14]. Solutions tailored to function in very restrictive settings, tests lacking robustness, and the limited involvement of end users were emphasized as major limitations of the existing ETA research initiatives [11,12]. A tradeoff between the accuracy and costs of developing and deploying an indoor navigation solution was highlighted as a limiting factor after a thorough review of various technologies. Wi-Fi was pointed out as the most economically feasible alternative as long as the users can tolerate lower accuracy [15]. The aforementioned reviews provide indications for general research directions in the field; however, these trends are often limited to the insights and assumptions made by the academics and often underestimate the feedback and needs of end users [11,12].

The aforementioned reviews identify trends in recent indoor navigation technologies and sensor-based hardware. However, they mostly deal with indoor navigation technologies without careful consideration of blind individuals’ needs [6,16]. We assume that it is necessary to take into account blind experts’ opinions of such systems. For this reason, publications from the last five years, dedicated to the description of indoor navigation systems for VI persons, were selected in this review. In addition, a semi-structured survey of VI experts was conducted. Due to the new technological advances in this rapidly evolving area, particular attention is paid to the interfaces and image processing subsystems.

Hence, after discussions with blind experts about ETA devices and technologies, and after conducting market demand research, it was decided to review current research on indoor navigation solutions. To aggregate specific details about the most significant issues and problems faced by blind and VI persons, an online survey was developed and conducted. The analysis of the survey results provided a basic understanding of users’ expectations and requirements for indoor ETA solutions. It enabled the identification of some new developments in the field.

The specifics of the method employed in this paper distinguish it from the existing reviews in the field. Our focus on the hardware part of ETAs and mapping the properties of existing solutions to the end user needs and expectations provides new perspectives that were not discussed in research publications before. In addition, we put emphasis on camera-based ETAs, utilizing novel computer vision algorithms and requiring no adaptation of the environment to function. A combination of literature review and user surveys enables a quantitative and qualitative evaluation of the existing ETA systems using criteria of the highest importance to the users.

This article is organized as follows. Section 2 provides a review of indoor navigation systems dedicated to VI persons. In Section 3, the results of the semi-structured survey conducted with blind experts are presented. Section 4 presents the results of the qualitative evaluation, and Section 5 includes a discussion and brief conclusion.

## 2. Existing Indoor Navigation Systems

The review included scientific publications that describe systems for indoor navigation and cover various indoor navigation technologies. Publications from a period of approximately five years (2014–2019) were included. The IEEEexplore, ScienceDirect, PubMed, and ACM DL databases were queried using combinations of the keywords “visually impaired”, “blind”, and “indoor navigation” (exact query: (“visually impaired” OR blind) AND “indoor navigation”). The search found 226 publications that matched the query. Publication abstracts were reviewed to exclude irrelevant papers. A full-text analysis identified 27 papers that met the inclusion criteria, such as including a description of indoor navigation with various indoor navigation technologies. The review was not limited to complete and functioning system prototypes, but it also included frameworks and technical descriptions of indoor navigation systems. 

**Figure 1:**
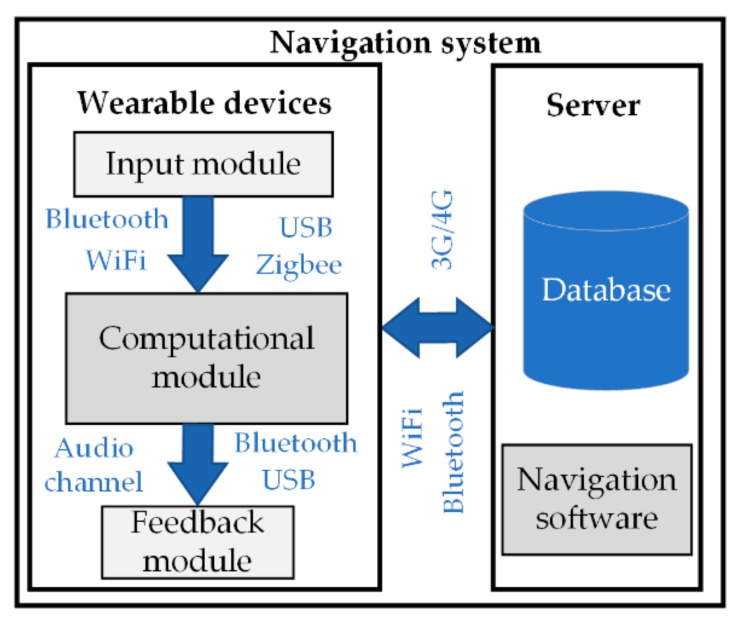
Indoor navigation system block diagram (the communication technologies used most often in all reviewed prototypes are indicated in blue).

Indoor navigation systems (Figure 1) generally consist of two parts: wearable devices and a server.

This review is mainly focused on the wearable device component of navigation systems. These devices are often complex systems, combining at least three types of modules: an input module, computational module, and feedback module. The findings of this review provide an overview of these three module groups.

### 2.1. Input Module

Input modules used in indoor navigation systems can be enumerated into three groups according to the sensors used for navigation: non-camera-based, camera-based, and hybrid. Non-camera-based systems use various sensors to sense the environment and represent it to the user. Camera-based systems use a live camera stream or images as the main source of information to represent the surroundings. Hybrid systems combine both input types.

#### 2.1.1. Non-Camera-Based Systems

Non-camera-based systems are common in industrial settings and are often used for robot navigation [17,18,19,20]. Industrial indoor navigation systems use sensor network tags (Radio-Frequency Identification (RFID), Near-Field Communication (NFC), Bluetooth Low Energy (BLE), and Ultra-Wideband (UWB)) for more rapid identification of various items in large warehouses. Wi-Fi and magnetic-field based methods are often suggested for localization indoors. It has been proposed to adopt these technologies to solve the problem of indoor navigation for VI persons. To be trackable, VI users of non-camera-based systems must carry the dedicated equipment (tag or tag reader, smartphone, or other receiver/transmitter device) while moving in their surroundings. The electronic tags have information that is used for additional information lookup in a database and location of the user on the environment map. The number of tags required to use indoor navigation depends on the selected sensor’s technology. For example, RFID tags can be active (operation range ≤ 40 m to ≤ 1 km [21]) or passive (operation range ≤ 0.5 m to ≤ 10 m [22]), and their count depends on the exact case requirements for accuracy. Active RFID tags have a much wider operating range. However, they need batteries, which requires additional maintenance for such systems.

Systems using NFC [23] tags do not require a separate scanner, since the scanner is built-in and available in various devices. It provides high accuracy. However, due to the relatively small operating distance (up to several centimeters), the detection of environment-integrated tags for VI persons could be challenging.

Bluetooth Low Energy (BLE) is another technology that can be used to create a network of navigational tags [24]. In comparison to NFC, it has a wider operating range (up to 75 m) and requires fewer tags to cover a certain area. It reaches an accuracy of 1–2 m, depending on the signal spread pattern. The advantage of BLE-based systems is that indoor wayfinding for VI persons does not require constant knowledge of the user’s location. It is necessary to direct the user more accurately at meaningful indoor space points (example stairs, elevators, office doors, etc.) [24].

Ultra-Wideband (UWB) technology for indoor localization is relatively accurate (± 0.15 m, 95% confidence intervals to determine the position of the user) and does not require direct visibility between the tags and the sensors. UWB signals are commonly used for positioning and orientation. This technology has been used to create a SUGAR [25] system (an indoor navigation system for visually impaired people which uses UWB for positioning) that helps VI people navigate indoors. UWB sensors have an operating range of 90 m at low-data-transfer mode, making the technology ideal for deployment in large buildings. 

The authors also present an algorithm for calculating the position of a user wearing a receiver within the grid. For instance, one of the first and most widely recognized infrared (IR) indoor positioning systems is the “Active Badge” system [19] for finding people in a room. The users wear “Active Badges” that emit short IR pulses, containing unique identifiers with a frequency of 0.07 Hz. The signal is collected by a network of stationary IR receivers inside a building and used for localization. Following this principle, Islam et al. [26] built a grid of 16 IR transmitters that continuously monitor the user’s position and deployed this grid in an indoor environment. Unfortunately, the proposed algorithm does not scale well due to the memory required for computing several paths simultaneously in larger grids. 

IR sensors for indoor navigation are typically used to determine the distance from an obstacle [27]. The operating range of IR-based positioning can reach 0.2–14 m. For example, Time-of-Flight (ToF) IR sensors measure distance by emitting pulses of eye-safe IR light and recording the time it takes for the light to return to the sensor [28]. The sensors measure distances up to 14 m with 0.005 m resolution and 0.04 m accuracy. IR systems require direct visibility between the sensors. A short operating distance makes IR-sensor-based positioning systems complex and inefficient in comparison to other technologies.

One of the less common methods for indoor navigation uses magnetic field for localization. This approach utilizes the magnetic landscape of buildings, created by internal constructions, such as steel and other materials, interacting with the magnetic field of the Earth. While the natural magnetic field is relatively uniform, ferromagnetic materials used in modern buildings (steel elements, doors, elevators, etc.) create disturbances that shape the magnetic landscape of a specific building. Such distortions generate anomalies when a magnetic field is used to determine the direction [29]. Zhang et al. propose GROPING, an all-in-one system that includes map generation, localization, and navigation [30]. GROPING relies on the geomagnetic field to characterize indoor locations. The solution combines two main features: magnetic fingerprinting and lightweight localization. A combination of these features utilizes crowdsensing for magnetic fingerprinting, which is later used for constructing floor maps from an arbitrary set of walking trajectories. After the map is constructed, live magnetic field measurements are used for estimating the location of the user in the fingerprint map [30]. Such magnetic field-based systems [29,30,31] do not require additional equipment on the user side. An ordinary smartphone containing an integrated accelerometer, gyroscope, and magnetometer are sufficient to register changes in the magnetic field and perform the calculation required for localization and navigation. Unfortunately, we have not identified any ETA tailored for VI individuals using this technology.

Several approaches have been used for utilizing Wi-Fi hotspots for indoor localization and navigation. Received Signal Strength Indicator (RSSI) localization methods are based on measuring the signal strength from a client device to several different access points and calculating the location of the user using triangulation techniques. An extension of RSSI is a fingerprint-based method. Similar to the magnetic field-based localization, a Wi-Fi fingerprint landscape is created beforehand and labeled with additional details required for localization. While the RSSI method estimates the position using triangulation in real-time, a fingerprint-based approach gets location information from a landscape database by fingerprint matching [32]. RSSI-based Wi-Fi systems provide a median accuracy of ± 0.6 m and suffer from dynamic obstacles interacting with radio propagation [33]. To increase the efficiency and accuracy of these methods, Ashraf et al. [34] proposed a Wi-Fi fingerprinting approach that exploits the uniqueness of the Wi-Fi coverage area and overlaps of the coverage areas of several access points (APs). Experimental results demonstrate that the method can mitigate the influence of device diversity and minimize the size of the fingerprint database, thus improving the efficiency of localization [34].

Non-camera-based technologies used to build indoor navigation tools for the blind have their advantages and limitations. The choice of technology is influenced by many factors that characterize the use case of interest. From a technical perspective, UWB may be the preferred choice, as it maximizes accuracy. However, the cost of deploying the infrastructure and the short battery life of the navigational tags require compromises on accuracy and operating range. BLE is less accurate than UWB, but it is more flexible and easier to deploy. RFID tags are cheaper than UWB, and the overall cost of the receivers and technology is similar to that of UWB. IR is rarely used for indoor navigation systems. IR-based systems struggle to detect transparent objects such as windows or glass walls. Wi-Fi or magnetic field-based technologies for indoor localization are rather attractive. Wi-Fi is already widely available inside the buildings; magnetic field-based localization does not require additional infrastructure to be deployed. However, the limitations of these technologies should be taken into consideration. The accuracy of Wi-Fi systems is influenced by a diversity of client devices, temporal changes of surroundings, and the use of building materials interacting with radio signals. Magnetic field-based technology has certain disadvantages such as changes in the value of magnetic field vector depending on the position and orientation of the client device walking speed and even the level at which the device is held.

#### 2.1.2. Camera-Based Systems

Camera-based systems use video cameras as their main source of information. Such systems are also capable of object recognition in addition to navigation functionality. Depending on the type of camera sensor, the input can be divided into the following categories: single camera (charge-coupled devices (CCD) or complementary metal–oxide–semiconductor (CMOS) image sensor [35]) and 3D camera.

In single-camera systems [36,37,38,39], cameras without depth sensors are used. Building on ideas from non-vision-based systems, the use of fiducial markers (Aruco, QR codes) is suggested [37,38] as an alternative to computationally intensive video analysis. Markers simplify complex video processing, as the system focuses on the recognition of a finite set of markers integrated in the environment. Idrees et al. [37] tested the recognition of QR codes under varying light conditions and markers of different sizes and blurriness levels. QR codes were easily detected under low light conditions and at a blurriness ratio of up to 60%. Manlises et al. [39] demonstrated the feasibility of obstacle and shortest route detection using a single web camera without compute-intensive hardware. The authors state that VI persons were able reach their chosen destination. The post-survey of results showed that the efficiency of such a system can reach 80% by implementing CAMShift [40] and D* [41] algorithms.

In the case of 3D cameras, indoor navigation systems can be divided into ToF camera-based and depth sensor (RGB-D) camera-based. Both are capable of detecting objects/obstacles and estimating the distance to them. Regardless of the camera type, the systems work in a similar manner. Localization of the user’s position in the room is estimated by visual odometry using RGB-D or ToF cameras.

In ToF cameras [42,43,44], an IR ToF sensor is used. It illuminates the environment with modulated infrared light and measures distances up to 5 m with an accuracy of ±0.01 m. For pose estimation, the 3D range data of the ToF camera are registered to form a large 3D point cloud map. Ye et al. [42] proposed a new indoor localization method using a ToF camera that outperformed the benchmarks. It obtained a more accurate pose more quickly than the state-of-the-art plane-based simultaneous localization and mapping (SLAM) methods. Ye et al. [43] proposed a system with a ToF camera used for both pose estimation and object recognition in an unknown indoor environment. Such a system has a success rate of over 86.7% for object recognition using a Gaussian mixture model (GMM)-based method. The pose graph algorithm used for pose estimation works with greatly reduced final position errors compared to a dead reckoning method. In Jeon et al. [44], a ToF camera was employed as the main sensor in the proposed system, since it is small in size and requires relatively low computational power. This may result in better pose estimation and object recognition performance. Such a system enables wayfinding and obstacle avoidance by using a single 3D imaging sensor for human-intent detection, pose estimation, and 3D object recognition. The uniqueness of this system compared to the others in the review is a fabricated vision processor in the 40 nm CMOS process that helps successfully detect obstacles and calculates safe distances in multiple directions while correcting for the camera position based on the posture data from the inertial measurement unit (IMU).

An RGB-D camera [45,46,47,48,49,50,51,52] uses a color sensor that takes RGB values and an IR depth sensor that takes image depth data with high accuracy. IR light is used for depth data collection. The measures range to 10 m, but they vary depending on performance accuracy, scene, and light conditions. Yang et al. [46] showed that using two RGB-D cameras on different body levels (one on the head and a second at the waist) enables users to take the navigable direction and pay attention to potential hazards near their feet. The results suggest that the safety and robustness of navigation is enhanced by combining the perception of the environment in the direction of movement with low-lying obstacle detection. Zhang et al. [49] proposed an indoor navigation system that uses a camera for floor plan capturing and an RGB-D camera for user localization and corner and wall detection. By matching the detected landmarks to the corresponding features on the digitalized floor map, the system localizes the user and provides verbal instruction to guide the user to the desired destination. The errors in localization are within 0.2 m in most cases, which is accurate enough for navigation. Similarly, as in the previous study, Xiao et al. [52] proposed a technique to determine location in two ways: one without odometry, which recognizes the current view of the user based on visual landmarks captured by a camera; and another using an RGB-D camera, which computes the relative pose between the current and previous pose of the user based on odometry metrics. The authors noticed that in a real-life scenario using a head-mounted camera, it is challenging to compensate for the blur effect caused by the motion of a human body.

#### 2.1.3. Hybrid Systems

Hybrid indoor navigation systems typically consist of navigational tags of a selected technology and an image processing device. Nair et al. [53] presented a hybrid navigation system using a user-held Google Tango (https://www.businessinsider.com/google-tango-2017-5?r=US&IR=T#in-this-demo-tango-is-used-in-a-classroom-to-show-a-bunch-of-students-a-virtual-globe-floating-in-the-middle-of-the-room-2) device for video processing and BLE for localization. This device is designed to create a 2D plan for each floor using the affine transformation function from the OpenCV library. Another system, presented by Simoes et al. [54], uses an RGB camera, radio frequency (RF), and visual markers for improved localization. Stationary environment-deployed cameras can be used for video capturing [55], as presented by Dao et al. Wi-Fi access points here are used as beacons for improving localization. The authors noticed that the use of different technologies boosts the accuracy of the overall system. Nair et al. [53] noticed that users perceived the hybrid system inputs as better than the standalone BLE systems, even though the BLE system was equally successful in guiding users to a destination quickly, safely, and with little assistance from the researchers.

### 2.2. Computational Module

Hardware used for the development of navigation systems can be grouped into off-the-shelf communication devices, computational devices, and mixed. Off-the-shelf communication devices (e.g., smartphones) offer a well-functioning ecosystem hosting many of the required sensors and communication elements. Computational devices (for example Arduino, Raspberry Pi) are often chosen with the aim of extending the functionality of existing off-the-shelf units when they are not suitable for a certain use case. 

Since most of the systems described in the studies under review are in the development phase, personal computers are usually used for data processing, storage, and the realization of complex navigation or video processing algorithms [21]. Off-the-shelf communication devices such as smartphones [37,45,53] or tablets [42,45] are usually used because they provide a convenient means of addressing the research challenges. These devices ensure standardized data transfer (over Bluetooth, Wi-Fi, 3G/4G) between wearable devices and servers. They are equipped with an IMU, a pedometer, and infrared sensors. All these sensors and an integrated camera or 3D camera enable the development of new applications to solve various problems, or the use of existing systems to do the same.

Computational devices, such as application-oriented integrated circuits (ASIC), microcontrollers, or minicomputers are preferred when sensors and cameras that are not available in off-the-shelf devices are required. Low-cost microcontroller (Arduino or ARM-based) systems [26,28,43,56] are particularly attractive in the initial development phases. If the task requires more computational resources and flexibility [22,39,57], Raspberry Pi (https://www.raspberrypi.org/) systems are preferred. They allow implementations in the Python programming language. In the final stages of development, an ASIC is produced to address the requirements of a functional prototype [44]. It enables optimization of the system’s size and energy consumption, but is usually time-consuming.

In some cases, both types of devices are used to enhance performance. For example, a smartphone may be used for data exchange with a computational device and a user feedback device [56]. Occasionally, a minicomputer can be used to transfer video data to the server for further processing [39].

### 2.3. Feedback Module

The processed environment representation is transmitted to the user using one of three channels: sound, touch, or image. Each of these methods has its own advantages and disadvantages.

VI persons rely primarily on hearing for environment perception. Many of the papers included in this review selected this channel as an interface between the user and the system. In many cases, use is made of ordinary headphones, which completely cover the ears and prevent perception of the environment. Alternative solutions, which ensure the ability to sense the environment and receive feedback from the system simultaneously, are also considered by researchers, and take the form of specially designed mini headphones and bone conductive headphones. 

Tactile transmission has long been used as the main way to transfer received and processed information from a transmitter [58]. The original and simplest tactile aid for VI persons is the white cane. Usually, it has only one function: to identify obstacles close to the user. There have been many attempts to enhance the functionality of the white cane using technological aids [24,44,45], for instance by adding vibrating motors or voice (audio) guidance functionality. Guidance by vibrating motors is realized by placing these motors directly on the cane, and voice commands are transferred by headphones. In addition, vibration guidance can be implemented on a belt, with vibrating motors that can inform the user about obstacles alongside them or at the level of their body height [28,48,52,53].

The transmission of information using images (so-called smart magnifiers) is designed to enhance the visual capabilities of the user or to broaden the field of vision of persons with tunnel vision impairment. Augmented reality (AR), in which the user is shown guiding cues, has been proposed as a potential solution [50]. 

Table 1 summarizes the papers included in the review.

The selected publications show that authors are increasingly focusing on image-processing-based systems (see Table 1). This development has been influenced by technological improvements in the field of image processing software, improvement of 3D cameras, and improved hardware performance and mobility. Despite the fact that this review is focused on indoor navigation systems, solutions with a wider range of functionality and the ability to function outdoors may be more appealing for the blind.

The majority of camera-based solutions use RGB-D sensors, because depth sensing is crucial for 3D reconstruction and scene understanding. Active depth sensors provide dense metric measurements but are often subject to constraints such as a limited operating range, low spatial resolution, sensor interference, and high power consumption. However, recently, systems with RGB-D sensors have become popular due to their compactness. In addition, the smartphone market continues to expand its offering of image capture systems containing depth sensors. Using devices existing in the market helps to reduce the costs of development and manufacturing. It should be noted that object detection is the most usable function for VI persons in various indoor navigation systems (see Table 1). However, only seven reviewed systems are capable of object recognition. These systems are only camera-based. 

Various types of hardware are used for the development of navigation systems (Table 1). In indoor navigation systems, smartphones are used together with other devices such as laptops or personal computers. In these cases, a smartphone was used as a data transmission channel for sending information to a personal computer. Furthermore, laptops and personal computers were used as computational and storage devices. Computational modules such as microcontrollers and minicomputers were used for more flexible tasks. Most solutions involve wireless communication tools (Bluetooth, Wi-Fi, Zigbee, etc.) for the transmission of information between nodes. These technologies could be used to assess the mobility and flexibility of the system. Audio transmission is a widely used mechanism for providing feedback between the system and the user.

## 3. Meta-Analysis: Survey of Blind Users’ Needs

This section reviews results relating to blind users’ defined needs. The intention is not only to highlight differences between technological deliveries on the one hand and expectations on the other, but also to perform an additional meta-evaluation of selected papers from the review. For this reason, a semi-structured survey of blind users was conducted. The survey helped to align end-user-defined main evaluation criteria and accordingly to assess selected research and development (R&D) prototypes for indoor navigation solutions. 

Twenty-five blind experts, most of whom reside in the EU region, participated in a semi-structured online survey and personal interviews. The main criterion for the blind experts’ selection was 10+ years of experience (or active interest) in the use of ETAs for the blind. The survey contained 39 questions covering demographic, sight-related, and open-ended questions regarding ETA systems. The survey was completed in February 2019.

Two VI individuals having long-lasting experience with ETAs were involved in designing the questionnaire. A series of focus group meetings and discussions allowed incorporating various perspectives of ETA use in daily life, which is important to the end users. A final version of the questionnaire was evaluated by the collaborating blind ETA experts. When performing the survey, 10 out of 25 blind experts were interviewed live, and recordings of the interviews were analyzed. Interviews provided additional insights, going beyond the questions of the survey.

The sample of expert users is characterized by a mean age of 33 years and includes mostly employed (52% fully employed, 20% partially employed) individuals, residing in big cities (84%). Sixty percent of the participants had higher education, and the average duration of professional work experience was 11 years. The experts were contacted following the recommendations of associations of blind and VI individuals. Their expertise level was double-checked using a number of control questions. Thus, interviewed blind experts well fitted for figuring out modern (technologically oriented) VI persons’ needs regarding preferred ETA characteristics. However, we did not have the intention to collect a representative survey of a whole worldwide blind population. The latter case would require a different survey approach, which is beyond the scope of this review paper.

**Figure 2:**
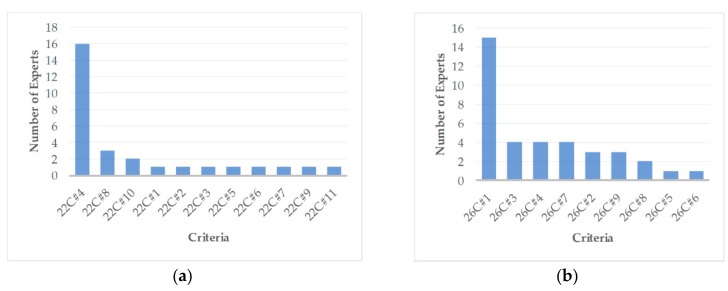
The importance of chosen criteria as defined by the respondents: (**a**) criteria ranked for the question, “What electronic travel aids (smartphone apps, navigation devices…) do you use for orientation/navigation indoors?”* (**b**) criteria ranked for the question “If you would consider creating a novel technological aid for navigation indoors, what functions would be most important to you?”^**^.

The use of open-ended questions made it possible to obtain more details regarding the preferences, suggestions, and actual needs for ETA solutions in outdoor and indoor environments. For instance, in the question, “What electronic travel aids (ETA) do you use for orientation/navigation outdoors?” 11 of the 25 respondents mentioned smartphone apps such as Trafi, Google Maps, Apple Maps, and other applications. Most respondents said that they use these ETA-enabling mobile apps because they fit their needs. Five respondents did not use any ETA devices. Different answers were obtained in the case of indoor orientation/navigation. For instance, 16 experts mentioned not using any ETA for indoor navigation (see Figure 2). In-depth interviews helped to figure out that this is due to the lack of commercially available suitable and convenient indoor ETA solutions. Thus, this market niche is still opened for R&D improvements. This argument we are going to explain below.

The differences between outdoor and indoor usage of smartphone apps are due to the absence of Global Positioning System (GPS) signals indoors, which impedes the usage of positioning and navigation apps. Thus, GPS-based apps are suitable for outdoor navigation and orientation, but they do not work well for indoor navigation. For this reason, researchers and engineers are searching for feasible indoor solutions. In this regard, there are a number of research directions for indoor navigation using Wi-Fi location approaches, visual data recognition algorithms, beacons, and RFID tags, among other technologies.

The diagram on the right (see Figure 2) makes clear an important observation (see 26C#1): blind experts prefer interactive tactile maps for navigation to distant destinations or chosen nearby objects (e.g., elevator, stairs, entrance, room numbers, exit, etc.). Another important observation is that blind experts prefer sound-guided navigation outdoors and tactile navigation indoors. 

In addition, the experts’ responses regarding the biggest indoor navigation problems were also surprising. For ranking these problems, each respondent was asked to list up to five critical problems in order of decreasing importance (with score 5 indicating the greatest importance and score 1 indicating the least). Subsequently, the 20 most common problems of highest importance were extracted (see Table 2). The importance scores were used to calculate the averages for each criterion—that is, each identified problem.

Average values were normalized and adjusted using weight coefficients, which took into consideration (1) the proportion of respondents (who selected that criterion) out of all respondents, and (2) the proportion of the sum of importance scores $S_{i}$ for the selected criterion i with respect to the sum of all scores for all criteria. This procedure reduced the bias in the estimates. Then, the weighting rate $W_{i}$ of each criterion i was calculated using Equation (1).

(1) $$W_{i}={\mathrm{Mean}}_{i}*\frac{n_{i}}{N}\frac{S_{i}}{\sum_{i}S_{i}},$$

where ${\mathrm{Mean}}_{i}$ denotes the mean importance score for criterion $i,n_{i}$ is the number of respondents who selected criterion i, and N is the total number of respondents (N=25).

As can be seen, the criterion ‘35C#3: Find room by number’ is the most important (weighting rate 0.642), followed by ‘35C#1: Find elevator’ (0.083), ‘35C#7: Read number in bank (0.056)’, ‘35C#2: Find stairs (0.056)’, and so forth. 

In sum, the blind experts’ survey indicates that compared to the commercially available outdoor solutions, there are not yet suitable indoor orientation/navigation solutions. Hence, there is a gap in the market for the development of suitable tactile and audio devices for indoor orientation/navigation.

## 4. Vision-Based Indoor Navigation Systems: Qualitative Evaluation

For the qualitative system analysis, the class of camera-based and hybrid indoor navigation systems was selected, because such systems are more versatile, require no installed indoor infrastructure, and were popular among interviewed blind experts. The survey also indicated that systems of this type meet well the expectations of VI persons.

For the qualitative evaluation, camera-based systems were selected, see Table 3. The survey results, together with insights from engineers, software developers, and researchers, made it possible to formulate criteria for the qualitative assessment that would make it easier to assess the suitability of the reviewed systems for VI persons. The formulated criteria are as follows:

The universality of the system is an important parameter. The system must have the following functions: image processing with an image database, the detection and identification of obstacles, the selection of route and the optimal path to the desired object, the transfer of information in tactile, acoustic, or visual mode, and operational indoors and outdoors. In addition to these features, the system should be easy to use, clear, and user-friendly. The value of the criterion is the number of system features listed above. If the function or feature is present in the system, 1 point is awarded. If not, 0 points are awarded. The maximum score for this criterion, corresponding to the ten system functions, is 10.

Object detection/recognition. For indoor navigation, it is important to inform the user of an impending obstacle in a timely manner. This is especially relevant in an unfamiliar environment. This criterion is distinguished even though the function is included in the system universality criterion. In our opinion, the detection/recognition of objects should be distinguished, as it provides an added value to the system. In addition, the survey showed that this is one of the most important functions for VI persons. Emerging obstacles would correct the trajectory of the movement, and in some cases, obstacles can even cause a change in the entire route. For example, when a user enters an unfamiliar environment and wants to find a certain office in it, a route is created. However, the floor plan does not mark the beginning of the floor repairing area, where there are various additional obstacles on the path or where the path is closed. Thus, the maximum score for this criterion is 2 for those systems that can detect and recognize objects (stairs, elevators, cabinet doors, tables, chairs, etc.) inside a building and read notes (names, numbers, etc.). 

Operation mode could be highlighted as important criteria for system evaluation. Most of the additional required and installed infrastructure elements for the indoor navigation systems are navigational tags (RFID, BLE, IR, or UWB, various markers, etc.). The building plan (with the tags) should be ready in advance. Two options for storage of the plan are available: it can be on a remote or a local computational device. When it is on a remote computational device, information is obtained only about the user’s location, and the route selection and movement trajectory are monitored remotely. Otherwise, everything is offline, because the processing device (i.e., the laptop), similar to the entire system, moves with the user. In this case, plans were created for one environment and do not renew. However, in some cases, this type of system can scan the plan and update the database. A third option is available when the system is operating in two modes: online (a plan with tags was created for reading from the server) and offline (routing and navigation is done locally). In systems with object detection, navigation is possible without any plan of the environment. Here, the user navigates their movement direction in order to avoid obstacles. Ideally, the system should operate independently of the location. It should have the feature of self-training when in an unfamiliar environment by collecting all possible environmental information (floor plan, layout of rooms and their numbers, plan of all possible tags, position of elevator and stairs, etc.), and it should be able to scan all available tags for creation of the desired route and direct the user along this route. The maximum score for this criterion is 3 points if the system operates in one of the described navigation modes and has additional infrastructure elements (RFID tag, NFC tag, markers, BLE, UBW, IR, IP Cam, etc.), and 1 for systems with a navigation plan or only with obstacle detection.

Feedback is a qualitative evaluation of the system that depends on the user’s needs and abilities. For example, voice commands are often unacceptable for VI persons while navigating indoors, because they wish the hearing organ (i.e., the ears) to be open for normal listening of the surrounding environment (several surveys have shown that voice commands are more desirable when navigating outdoors, however). Inside buildings, the tactile method for the transfer of commands or information is more acceptable. Admittedly, it has also some drawbacks—the tactile zones, which are affected continuously by tactile actuators, become numb after a while, and VI persons cannot understand all of the transmitted information. In contrast, visual information is only helpful to less visually impaired people. Thus, the maximum value of the criterion is 3 if all three ways of transmitting feedback information to the user are implemented in the system.

System evaluation was performed using Equation (2). The results are presented in Table 3.

(2) $$SE_{p}=\sum_{i=1}^{4}\frac{\sum_{j=1}^{m}c_{i,j}}{m},$$

where $SE_{p}$—system quality assessment, $c_{i}$—normalized evaluation criterion i of system, 

m—maximum possible score of criteria $c_{i}$.

**Figure 3:**
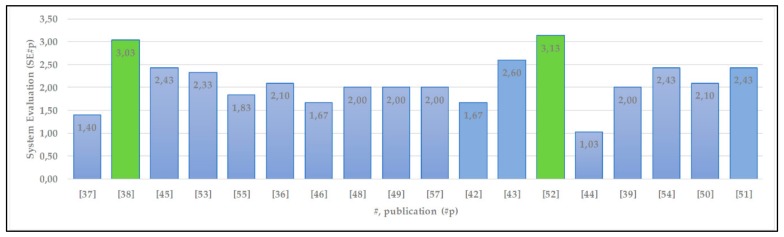
Results of qualitative evaluation of indoor navigation systems.

A summary of the qualitative assessment of the vision-based technology systems is presented in Figure 3.

The maximum qualitative value that a system could have received is *SE_p_* = 4. As can be seen in Figure 3, two reviewed systems (noted green columns) are closest to the maximum value. It should be noted that the majority (11 out of 18) of the image processing systems obtained 6 or even a higher score due to the versatility criteria, but none of them obtained a perfect score for this criterion. The highest qualitative value (3.13) is for Xiao et al.’s [52] system, which offers a complete set of technological solutions that can be used indoors and outdoors, but they only work online. The second-highest qualitative value of 3.03, with a 70% score for the versatility criterion, is for the [38] system, which can also work offline. However, it requires a computation device (e.g., a laptop) for image processing in real time.

## 5. Discussion and Conclusions

This article has presented an overview of indoor navigation systems for VI persons, as well as a qualitative assessment of these systems according to the criteria considered essential by blind experts. It should be noted that the review is not based on widely available commercial solutions. It is primarily concerned with prototype R&D published in recent scholarly papers.

The review indicates that many assistive devices for VI persons are in the active development stage. However, they mostly remain in the development stage or as prototypes only. Meanwhile, commercially available assistive devices are rarely used because they do not meet the needs and expectations of VI users (for instance, the devices are often too large, uncomfortable to wear, not reliable, too complicated, produce glitches). The survey of blind experts revealed that VI persons do not always trust new equipment or accept an interface.

In this regard, it was noticed that smartphones, as widely accepted ETA accessories, are acceptable to almost all VI persons, especially the younger ones. Thus, smartphone-based interface solutions are more likely to be accepted. The latest smartphones usually comprise various sensors, outdoor navigation aids, and audio and video processing capabilities. However, smartphones still do not have enough computational speed for high-flow real-time image processing. On the other hand, upcoming 5G-communication technology makes it possible to run image processing on a remote server-side.

After the discussions with the VI persons, we found that for distant orientation, they need to get basic information about important elements of environment in range of 5–20 m. Obstacles that are located at a distance of less than 2 m can easily be detected with the help of a white cane. The similar solutions have been found in the review [22,50,56,57,59,60], where the users are using ultrasonic sensors that are placed on white cane or on a user’s belt or jacket, and sensors operates in different directions with a range of up to 5 m. The authors of these solutions argue that such a distance is sufficient for decision making.

The study revealed that the choice of non-camera-based technology for indoor navigation and orientation is influenced by many factors that characterize the use case of interest. For instance, such equipment is limited to a specific location, as it must include a map of navigational tags (for navigation by Wi-Fi, BLE, UWB, NFC, Zigbee, etc.). This constraint dramatically reduces the universality, flexibility, and mobility of the system. The system becomes dependent on the sensors/tags laid out inside the building, which also require additional monitoring. Such systems would be more suitable for new buildings if the construction regulations were adjusted to anticipate the introduction of such tags. 

From a technical perspective, UWB may be the preferred choice for maximizing accuracy. However, the costs of deploying the infrastructure and short battery life of the navigational tags call for compromises on accuracy and operating range. BLE is less accurate than UWB, but it is more flexible and easier to deploy. RFID tags are cheaper than UWB, and the overall cost of the receivers and technology is similar to UWB. Meanwhile, IR is used for obstacle detection (except transparent objects such as windows or glass walls). However, IR usage for indoor navigation is rarely used. 

The review revealed that for vision-based indoor navigation, the popularity of 3D cameras with RGB-D sensors is rising. All proposed 3D cameras can detect objects/obstacles and estimate the distance and their location. However, for safer navigation, there are solutions with two such cameras or cameras with additional sensors for obstacle detection at different body levels. Single cameras require additional labels for object identification and databases for storing the captured view of the object’s location. Thus, in this case, flexibility is reduced.

Most camera-based technology solutions have suggested the use of ToF IR cameras. These cameras are large, but the latest solutions on the market are significantly reduced in size. Manufacturers of ToF cameras, such as PMD Technologies AG, together with Leica Camera AG [61], recently introduced a camera with a depth sensor using 940 nm wavelength, which works better than the prevailing 850 nm cameras. For instance, Samsung [62] showed improvements using a 940 nm structured light depth sensor.

The present study revealed that hybrid indoor navigation systems require more infrastructure elements, making them more price-sensitive than vision systems. However, the use of different technologies boosts the accuracy of the overall system. Users perceived hybrid systems as performing better than standalone BLE systems, although the BLE system was equally successful in guiding users to a destination.

Admittedly, the choice of sensors depends on the target goal of an application. For instance, ultrasonic sensors were a common choice in the reviewed studies for detecting obstacles (even transparent obstacles such as glass doors and windows) and determining distances. Laser sensors are highly accurate in the recognition of small objects, but the laser beam must be pointed exactly at the object, which is a difficult task for VI persons. It is important to note that IMU sensors increase the accuracy in the estimation of a user’s location and body orientation by refining the orientation error. Hence, the obvious solution is to use different sensors in order to improve the functionality. 

In sum, the results of the survey with blind experts, together with insights from engineers, software developers, and researchers, made it possible to formulate criteria for a qualitative assessment not only of the reviewed systems but also other systems as well. Based on these criteria, the qualitative value of the vision-based systems was calculated and compared.

## Figures and Tables

**Table:** 

* User-defined criteria for the question “What electronic travel aids (smartphone apps, navigation devices,…) do you use for orientation/navigation indoors?”: 22C#1-Aipoly Vision, 22C#2-Envision AI, 22C#3-Be my Eyes, 22C#4-don't use any device and app, 22C#5-don't work in the country, 22C#6-Brigo, 22C#7-Seeing Eye, 22C#8-white cane, 22C#9-Beacons option, 22C#10-phone (to enlarge the text), 22C#11-Google Maps.
** User-defined criteria for the question, “If you would consider creating a novel technological aid for navigation indoors, what functions would be the most important to you?”: 26C#1-interactive tactile map to the destination, including direction information (e.g., elevator, stairs, entrance, room numbers, exit, etc.); 26C#2-don't need; 26C#3-nearby objects recognition; 26C#4-text reading; 26C#5-photo reading; 26C#6-follow Bluetooth-labeled objects; 26C#7-above the head, left/right side, detectors for distance measurement; 26C#8-receipt ticket; 26C#9-find goods in the shop with tactile map.

**Table 1:** Summary of the papers included in the review.

# Publ.	Input	Sensors	Obstacle Detection/ Recognition	Feedback	Hardware	Working Area	Data Exchange
[21]	Non-camera	RFID	-/-	HP	RFID reader, Smartphone	In/Out	Bluetooth
[22]	Non-camera	RFID	+/-	H	RFID reader, RPi	In	ZigBee
[23]	Non-camera	NFC	-/-	HP	Smartphone	In	NFC, Wi-Fi
[37]	Camera	QR	-/-	AC	Smartphone	In	-
[59]	Non-camera	US	+/-	B	Arduino	In/Out	ZigBee
[25]	Non-camera	UWB	+/-	AC, B	Smartphone, PC	In	Wi-Fi
[38]	Camera	CAM, LS, IMU	+/+	AC	NPC, PC	In	-
[28]	Non-camera	IR ToF, IMU	+/-	VC	ARMuC	In/Out	Bluetooth
[45]	Camera	RGB-D, IMU	+/-	AC, VC	Smartphone (RGB-D)	In	Bluetooth
[53]	Hybrid	RGB-D, BLE, IR	+/-	VC	Smartphone, Tablet	In	Bluetooth
[55]	Hybrid	CAM, Wi-Fi	+/-	HP, VC	Smartphone, PC	In	Wi-Fi
[36]	Camera	CAM	+/-	AC	Smartphone, PC	In	Bluetooth, Wi-Fi
[46]	Camera	RGB-D, IMU	+/+	BHP	RGB_D pathfinder	In/Out	-
[48]	Camera	RGB-D, IMU	+/+	HP, VC	NPC	In	-
[49]	Camera	RGB-D, IMU	+/+	BHP	NPC	In	-
[57]	Camera	CAM, 2xUS	+/-	BHP	RPi	In	-
[42]	Camera	3D CAM	+/-	HP	Tablet, NPC	In	Wi-Fi
[56]	Non-camera	US	+/-	AC	Arduino, Smartphone	In	Bluetooth, Wi-Fi
[43]	Camera	CAM, 3D CAM, IMU	+/+	H, KP	ARMuC, Arduino	In	Wi-Fi, Bluetooth
[52]	Camera	RGB-D, IMU, CAM	+/+	BHP, VC	Smartphone, Tablet	In/Out	Wi-Fi, DSRC
[44]	Camera	3D CAM, IMU	+/-	VC	ToF camera, fabricated video ASIC	In	–
[39]	Camera	CAM	+/+	H	RPi, NPC	In	Wi-Fi
[24]	Non-camera	BLE	-/-	VC, B	Smartphone	In	Bluetooth
[54]	Hybrid	CAM, RF	+/-	BHP, VC	Arduino, RPi	In	Wi-Fi
[50]	Camera	RGB-D, US	+/-	HP, AR	Arduino, eCPU	In	-
[26]	Non-camera	IR, US	+/-	AC	Arduino Nano	In	-
[51]	Camera	RGB-D, IMU	+/-	AC, VC	NPC, Smartphone	In	Wi-Fi, USB

US-Ultrasonic Sensor, LS-Laser Sensor, IR-Infrared Sensor, CAM-CMOS Camera, DS-Distance Sensor, AC-Audio Command, AR-Augmented Reality, BHP-Bone Headphone, B-Buzzer, HP-Headphones, VC-Vibrotactile Command, H-Headset, KP- Keypad, NPC-Laptop, RPi-Raspberry Pi, ARMuC- ARM-Based Microcontroller, PC-Personal Computer, eCPU-Embedded CPU Board.

**Table 2:** A sorted list of the criteria defined by the blind experts while answering the question: “Please list up to five biggest problems (in diminishing order) you experience when orientating/navigating indoors (e.g., public places, at home, etc.)?”

No.	Criteria	*n_i_*	Mean*_i_*	Sum of Grades	Weighting Rate (*W_i_*)
1	35C#3: Find room by number	15	4.07	61	0.642
2	35C#1: Find elevator	6	3.67	22	0.083
3	35C#7: Read number in bank	5	3.60	18	0.056
4	35C#2: Find stairs	6	3.00	18	0.056
5	35C#17: Object recognition	3	4.67	14	0.034
6	35C#4: Find entrance	5	2.60	13	0.029
7	35C#11: Detect obstacles	4	3.25	13	0.029
8	35C#5: Find exit	6	1.83	11	0.021
9	35C#15: Steps and trip hazards	2	5.00	10	0.017
10	35C#8: Large open space	2	4.50	9	0.014
11	35C#12: Find goods in supermarket	3	2.67	8	0.011
12	35C#18: Text recognition	2	3.50	7	0.008
13	35C#10: Landmark in large space	1	5.00	5	0.004
14	35C#16: Find recycle bin	1	5.00	5	0.004
15	35C#19: Find corridor	1	5.00	5	0.004
16	35C#20: Find cash desk	2	2.50	5	0.004
17	35C#14: Other people	1	4.00	4	0.003
18	35C#9: Accurate distance to location	1	3.00	3	0.002
19	35C#6: Finding toilet (WC)	1	1.00	1	0.000
20	35C#13: find escalator	0	0.00	0	0.000
			**Total:**	232	

**Table 3:** Evaluation of camera-based and hybrid indoor navigation systems.

# Publication	Universality	Obstacle Detection/Recognition	Operation Modes	Feedback	Total Score	System Evaluation(SE)
[37]	4	0	2	1	7	1.40
[38]	7	2	3	1	13	3.03
[45]	6	1	2	2	11	2.43
[53]	5	1	3	1	10	2.33
[55]	5	0	2	2	9	1.83
[36]	6	1	2	1	10	2.10
[46]	7	2	1	1	8	1.67
[48]	6	2	1	2	9	2.00
[49]	5	1	2	1	9	2.00
[57]	5	1	2	1	9	2.00
[42]	5	1	1	1	8	1.67
[43]	6	2	1	2	11	2.60
[52]	8	2	2	2	14	3.13
[44]	2	1	1	0	4	1.03
[39]	6	2	2	1	9	2.00
[54]	6	1	2	2	11	2.43
[50]	6	1	1	2	10	2.10
[51]	6	1	2	2	11	2.43
